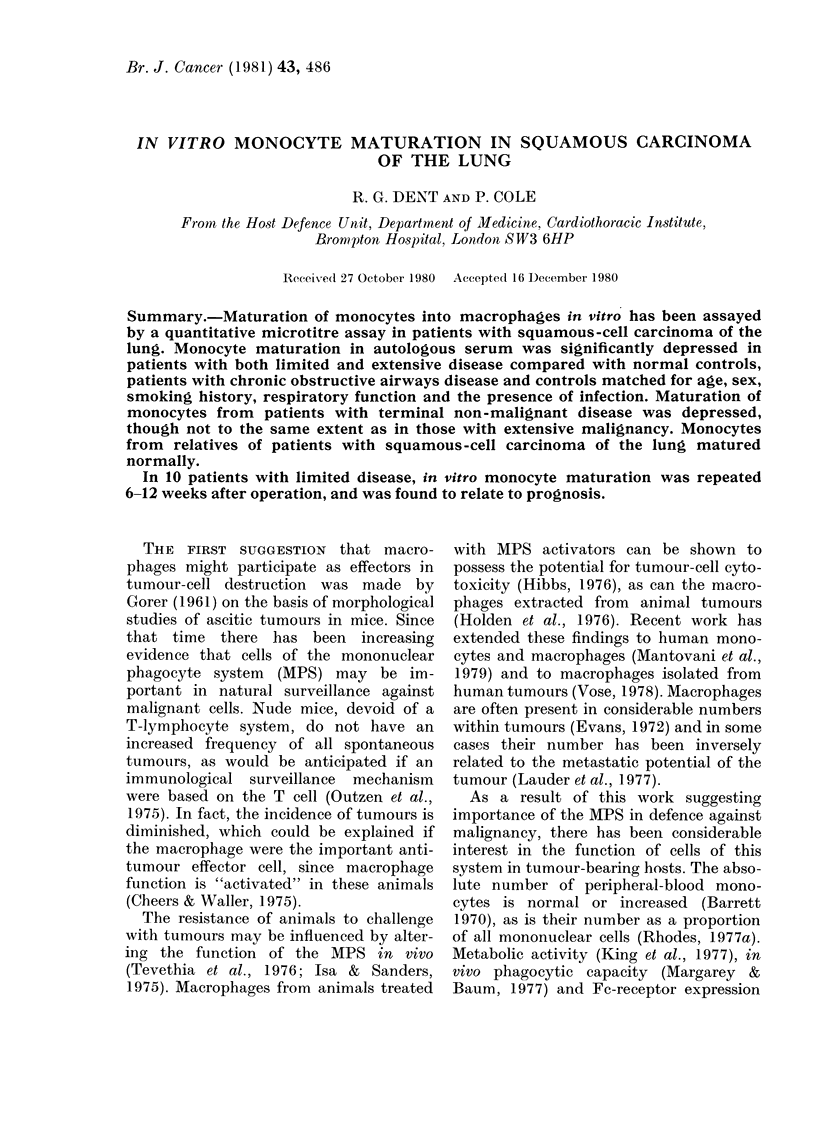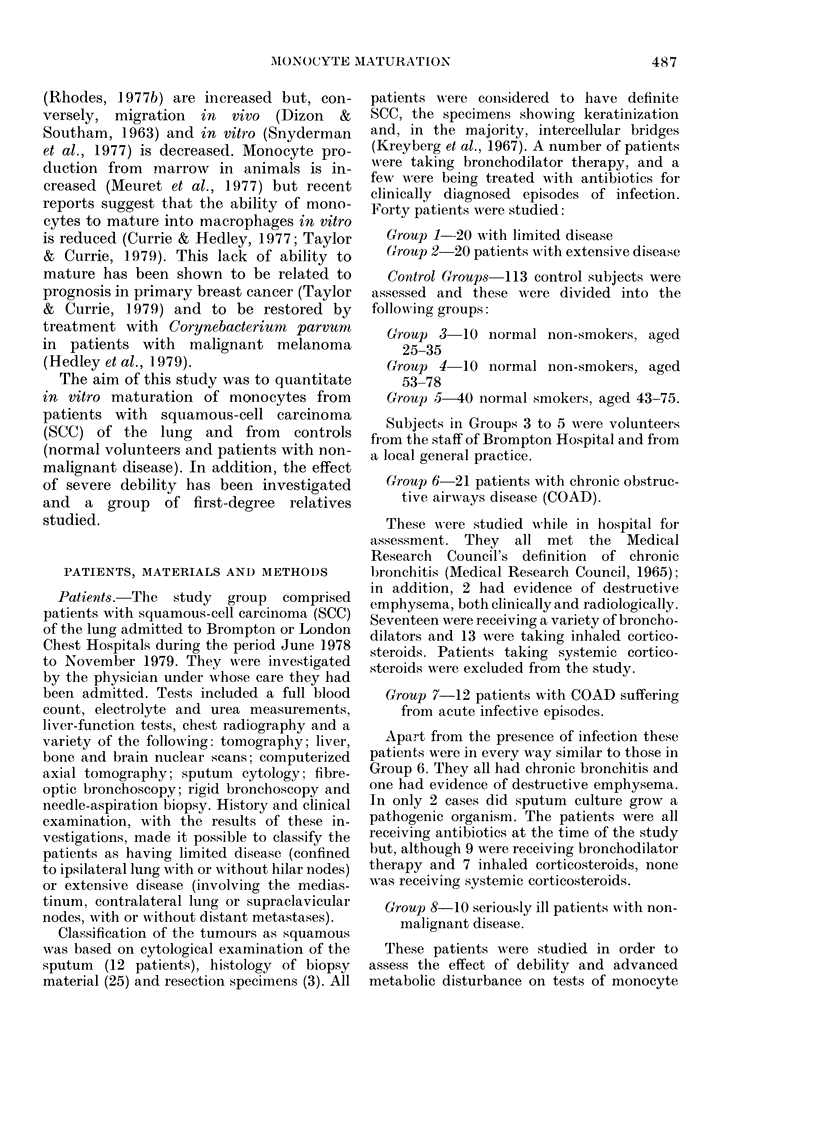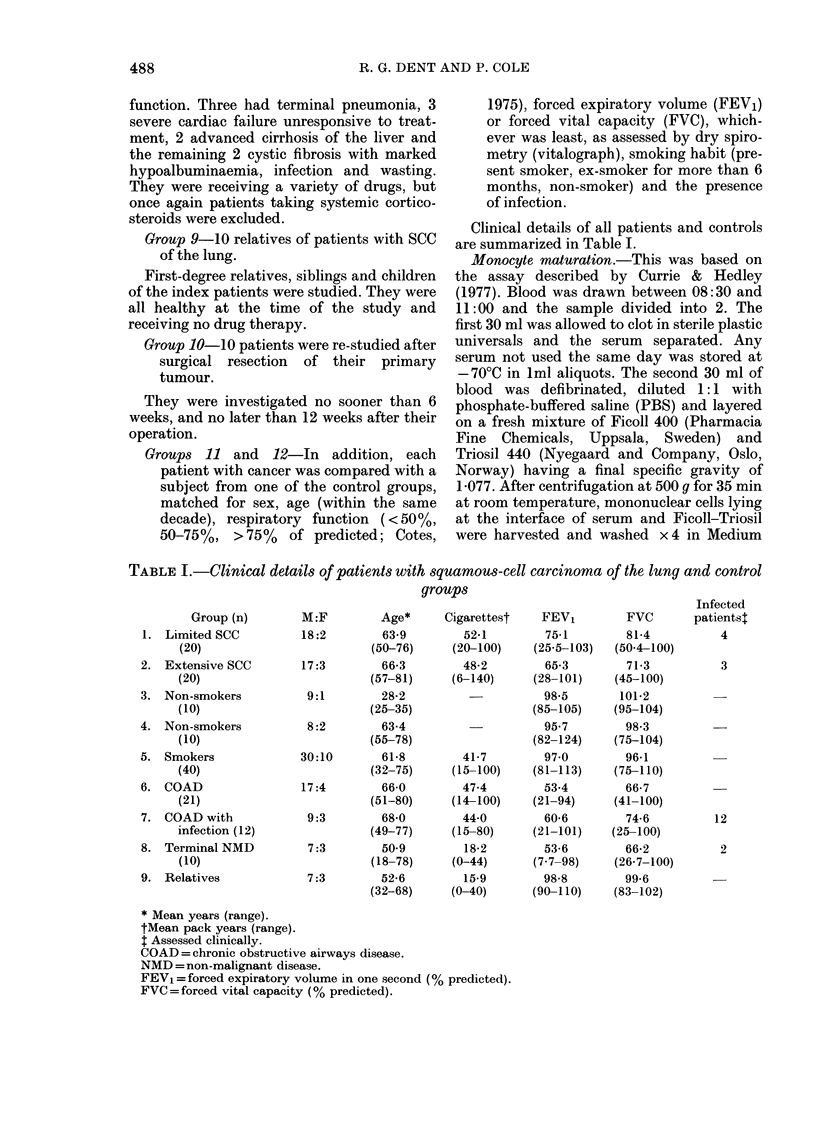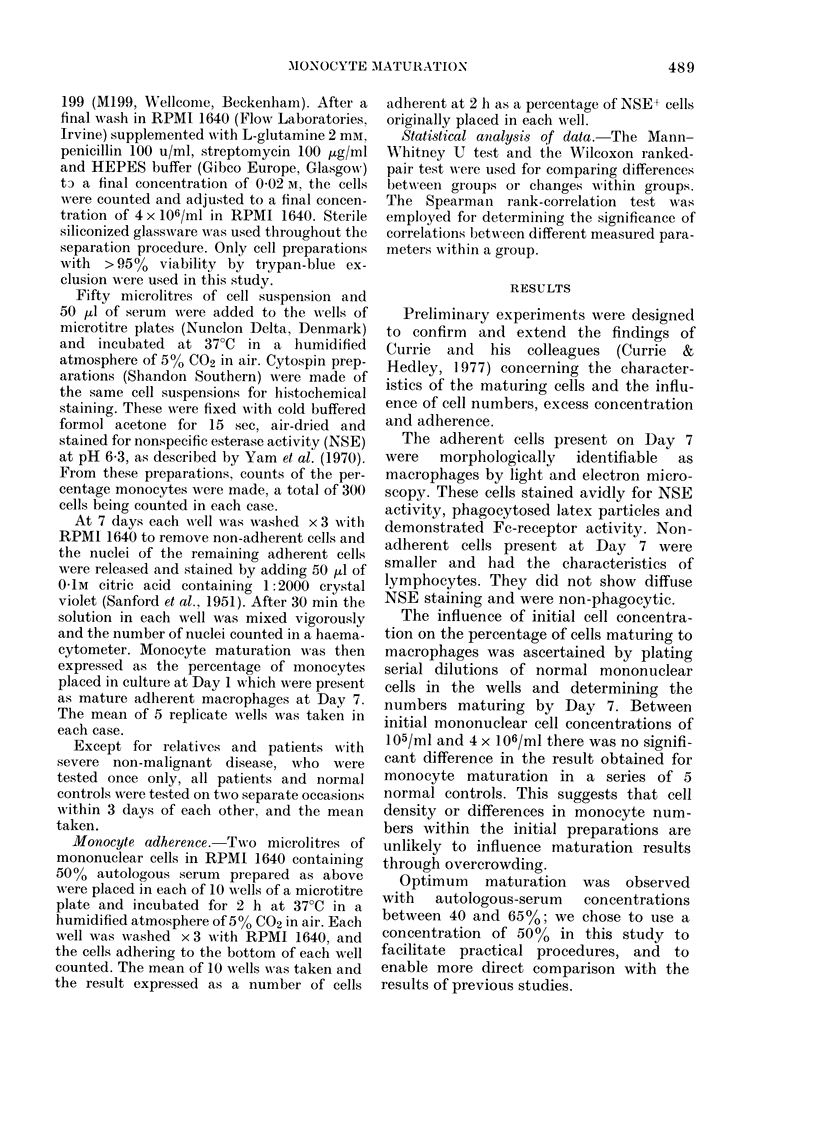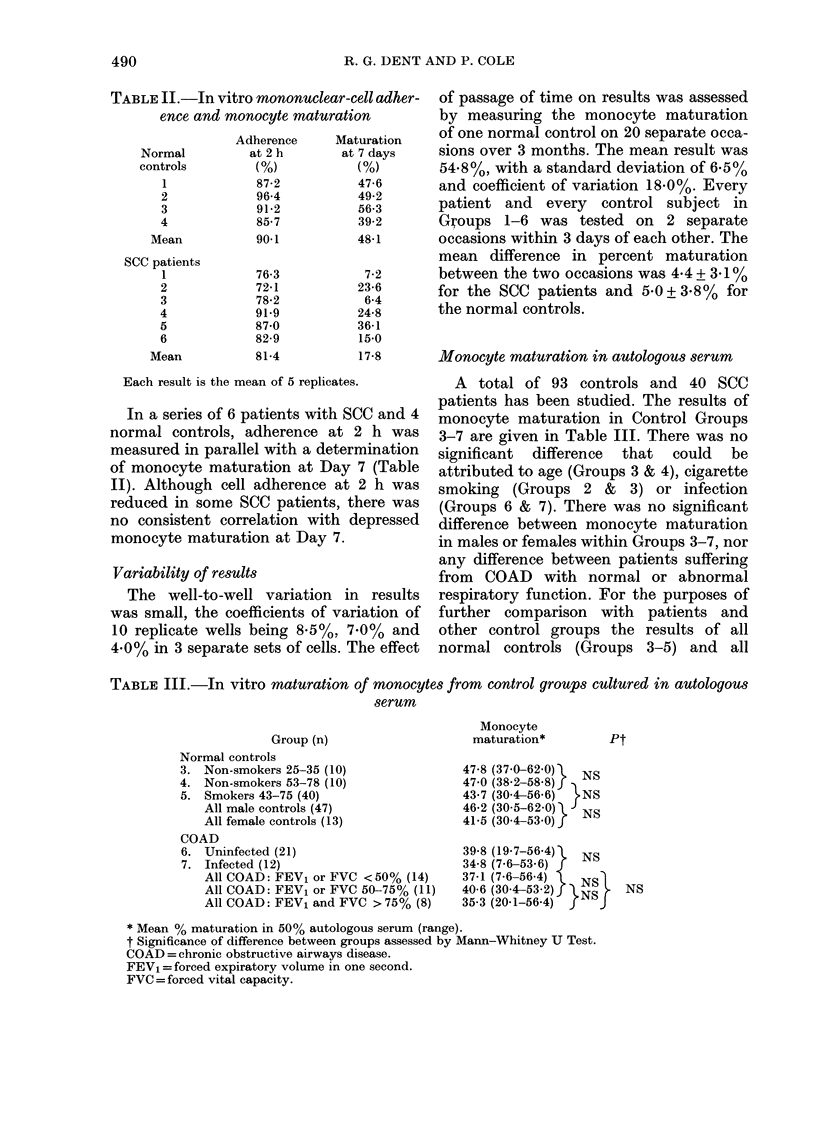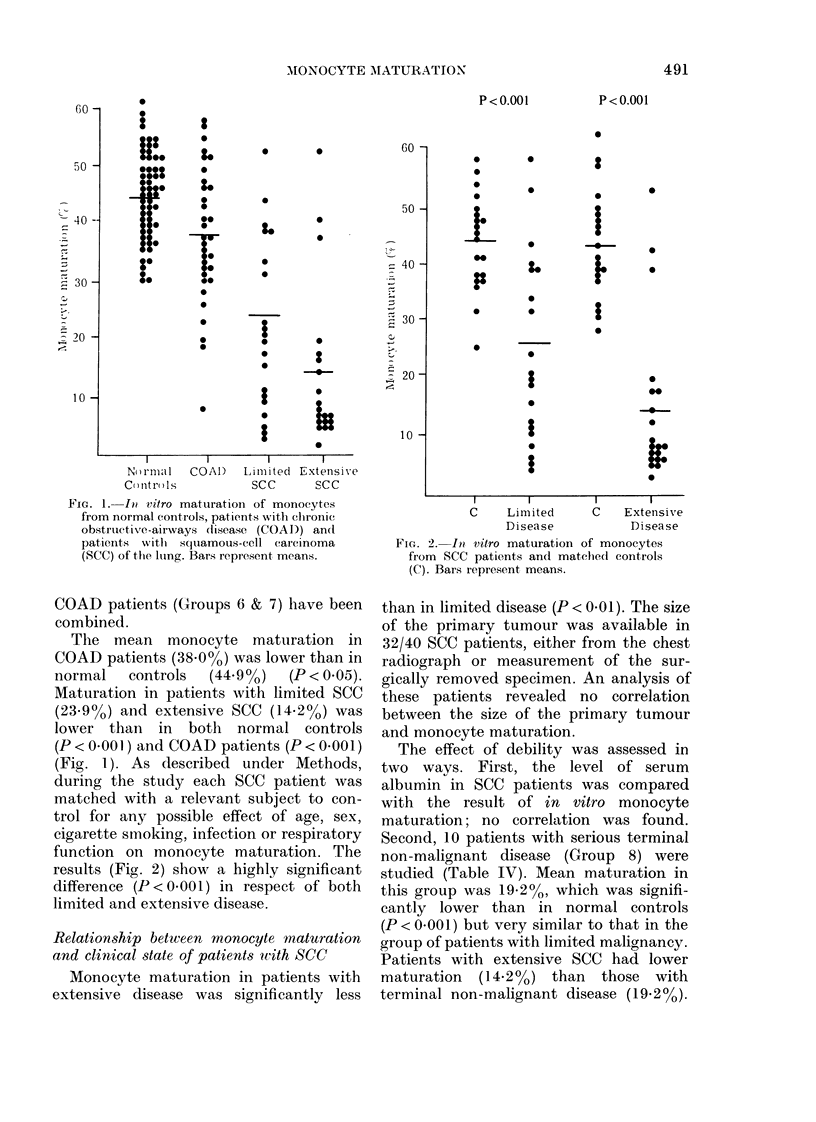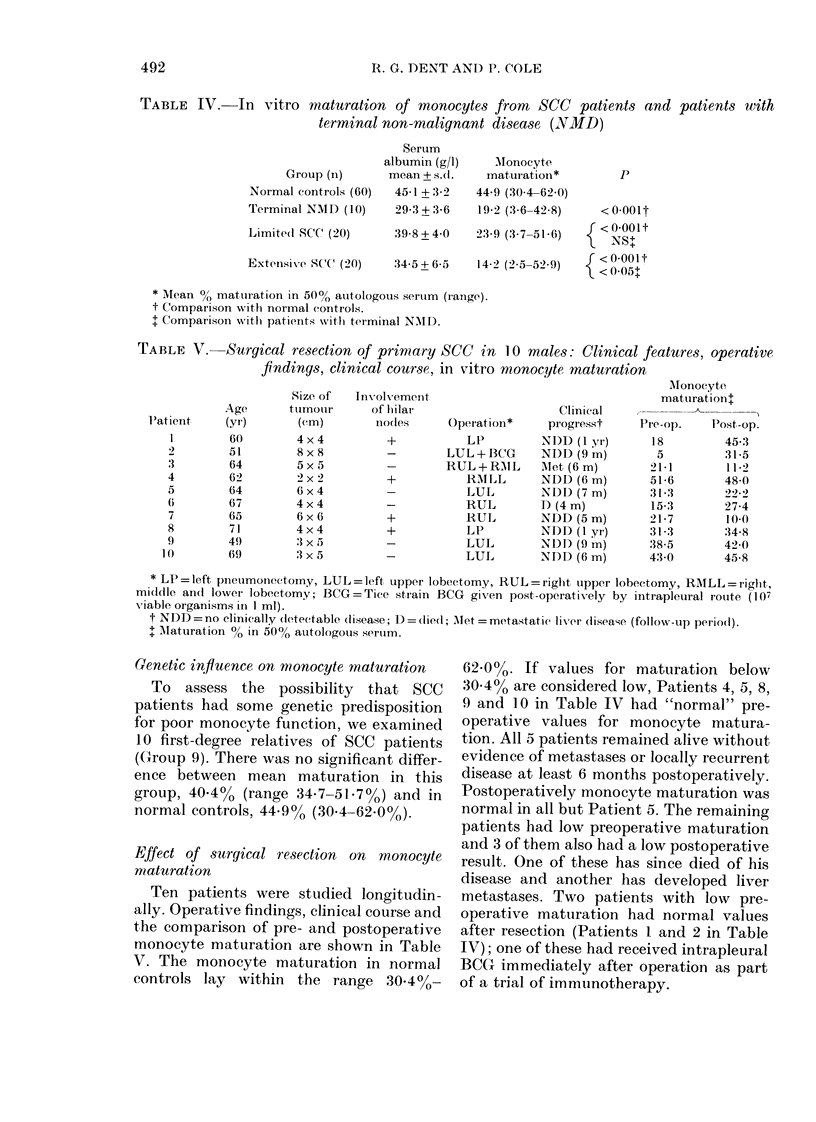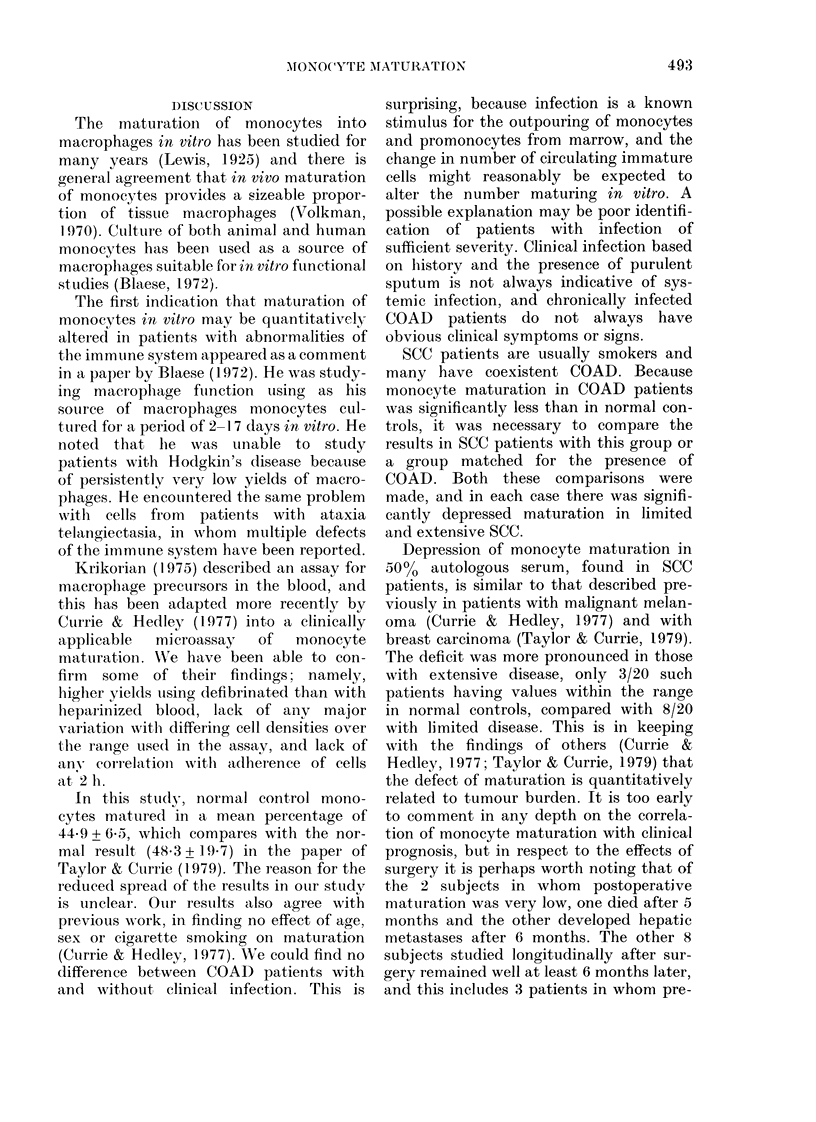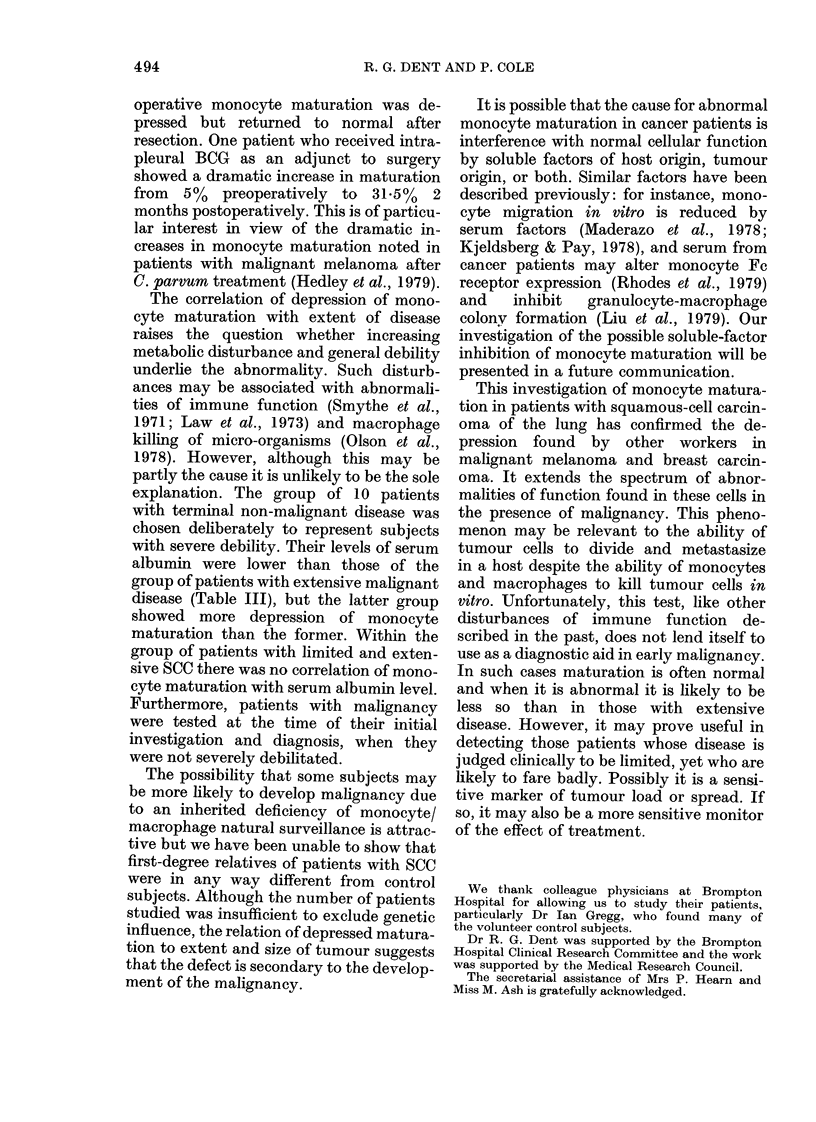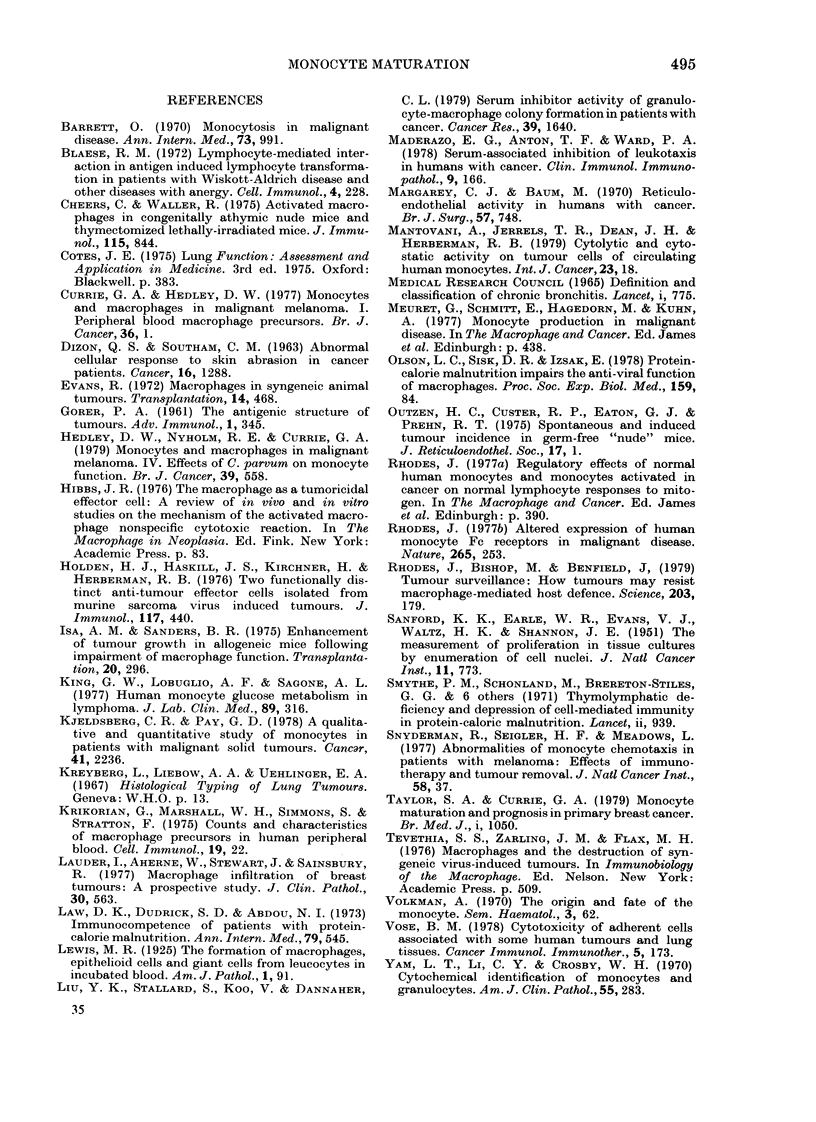# In vitro monocyte maturation in squamous carcinoma of the lung.

**DOI:** 10.1038/bjc.1981.71

**Published:** 1981-04

**Authors:** R. G. Dent, P. Cole

## Abstract

Maturation of monocytes into macrophages in vitro has been assayed by a quantitative microtitre assay in patients with squamous-cell carcinoma of the lung. Monocyte maturation in autologous serum was significantly depressed in patients with both limited and extensive disease compared with normal controls, patients with chronic obstructive airways disease and controls matched for age, sex, smoking history, respiratory function and the presence of infection. Maturation of monocytes from patients with terminal non-malignant disease was depressed, though not to the same extent as in those with extensive malignancy. Monocytes from relatives of patients with squamous-cell carcinoma of the lung matured normally. In 10 patients with limited disease, in vitro monocyte maturation was repeated 6-12 weeks after operation, and was found to relate to prognosis.


Br. J. Cancer (1981) **43**, 486

### *IN VITRO* MONOCYTE MATURATION IN SQUAMOUS CARCINOMA OF THE LUNG

R. G. DENT AND P. COLE

*From the Host Defence Unit, Department of Medicine, Cardiothoracic Institute, Brompton Hospital, London SW3 6HP*

Received 27 October 1980 Accepted 16 December 1980

**Summary.—Maturation of monocytes into macrophages *in vitro* has been assayed by a quantitative microtitre assay in patients with squamous-cell carcinoma of the lung. Monocyte maturation in autologous serum was significantly depressed in patients with both limited and extensive disease compared with normal controls, patients with chronic obstructive airways disease and controls matched for age, sex, smoking history, respiratory function and the presence of infection. Maturation of monocytes from patients with terminal non-malignant disease was depressed, though not to the same extent as in those with extensive malignancy. Monocytes from relatives of patients with squamous-cell carcinoma of the lung matured normally.**

**In 10 patients with limited disease, *in vitro* monocyte maturation was repeated 6–12 weeks after operation, and was found to relate to prognosis.**

THE FIRST SUGGESTION that macrophages might participate as effectors in tumour-cell destruction was made by Gorer (1961) on the basis of morphological studies of ascitic tumours in mice. Since that time there has been increasing evidence that cells of the mononuclear phagocyte system (MPS) may be important in natural surveillance against malignant cells. Nude mice, devoid of a T-lymphocyte system, do not have an increased frequency of all spontaneous tumours, as would be anticipated if an immunological surveillance mechanism were based on the T cell (Outzen *et al.*, 1975). In fact, the incidence of tumours is diminished, which could be explained if the macrophage were the important antitumour effector cell, since macrophage function is "activated" in these animals (Cheers & Waller, 1975).

The resistance of animals to challenge with tumours may be influenced by altering the function of the MPS *in vivo* (Tevethia *et al.*, 1976; Isa & Sanders, 1975). Macrophages from animals treated with MPS activators can be shown to possess the potential for tumour-cell cytotoxicity (Hibbs, 1976), as can the macrophages extracted from animal tumours (Holden *et al.*, 1976). Recent work has extended these findings to human monocytes and macrophages (Mantovani *et al.*, 1979) and to macrophages isolated from human tumours (Vose, 1978). Macrophages are often present in considerable numbers within tumours (Evans, 1972) and in some cases their number has been inversely related to the metastatic potential of the tumour (Lauder *et al.*, 1977).

As a result of this work suggesting importance of the MPS in defence against malignancy, there has been considerable interest in the function of cells of this system in tumour-bearing hosts. The absolute number of peripheral-blood monocytes is normal or increased (Barrett 1970), as is their number as a proportion of all mononuclear cells (Rhodes, 1977*a*). Metabolic activity (King *et al.*, 1977), *in vivo* phagocytic capacity (Margarey & Baum, 1977) and Fc-receptor expression

(Rhodes, 1977*b*) are increased but, conversely, migration *in vivo* (Dizon & Southam, 1963) and *in vitro* (Snyderman *et al.*, 1977) is decreased. Monocyte production from marrow in animals is increased (Meuret *et al.*, 1977) but recent reports suggest that the ability of monocytes to mature into macrophages *in vitro* is reduced (Currie & Hedley, 1977; Taylor & Currie, 1979). This lack of ability to mature has been shown to be related to prognosis in primary breast cancer (Taylor & Currie, 1979) and to be restored by treatment with *Corynebacterium parvum* in patients with malignant melanoma (Hedley *et al.*, 1979).

The aim of this study was to quantitate *in vitro* maturation of monocytes from patients with squamous-cell carcinoma (SCC) of the lung and from controls (normal volunteers and patients with non-malignant disease). In addition, the effect of severe debility has been investigated and a group of first-degree relatives studied.

### PATIENTS, MATERIALS AND METHODS

*Patients.*—The study group comprised patients with squamous-cell carcinoma (SCC) of the lung admitted to Brompton or London Chest Hospitals during the period June 1978 to November 1979. They were investigated by the physician under whose care they had been admitted. Tests included a full blood count, electrolyte and urea measurements, liver-function tests, chest radiography and a variety of the following: tomography; liver, bone and brain nuclear scans; computerized axial tomography; sputum cytology; fibre-optic bronchoscopy; rigid bronchoscopy and needle-aspiration biopsy. History and clinical examination, with the results of these investigations, made it possible to classify the patients as having limited disease (confined to ipsilateral lung with or without hilar nodes) or extensive disease (involving the mediastinum, contralateral lung or supraclavicular nodes, with or without distant metastases).

Classification of the tumours as squamous was based on cytological examination of the sputum (12 patients), histology of biopsy material (25) and resection specimens (3). All patients were considered to have definite SCC, the specimens showing keratinization and, in the majority, intercellular bridges (Kreyberg *et al.*, 1967). A number of patients were taking bronchodilator therapy, and a few were being treated with antibiotics for clinically diagnosed episodes of infection. Forty patients were studied:

*Group 1*—20 with limited disease
*Group 2*—20 patients with extensive disease

*Control Groups*—113 control subjects were assessed and these were divided into the following groups:

*Group 3*—10 normal non-smokers, aged 25–35
*Group 4*—10 normal non-smokers, aged 53–78
*Group 5*—40 normal smokers, aged 43–75.

Subjects in Groups 3 to 5 were volunteers from the staff of Brompton Hospital and from a local general practice.

*Group 6*—21 patients with chronic obstructive airways disease (COAD).

These were studied while in hospital for assessment. They all met the Medical Research Council's definition of chronic bronchitis (Medical Research Council, 1965); in addition, 2 had evidence of destructive emphysema, both clinically and radiologically. Seventeen were receiving a variety of bronchodilators and 13 were taking inhaled corticosteroids. Patients taking systemic corticosteroids were excluded from the study.

*Group 7*—12 patients with COAD suffering from acute infective episodes.

Apart from the presence of infection these patients were in every way similar to those in Group 6. They all had chronic bronchitis and one had evidence of destructive emphysema. In only 2 cases did sputum culture grow a pathogenic organism. The patients were all receiving antibiotics at the time of the study but, although 9 were receiving bronchodilator therapy and 7 inhaled corticosteroids, none was receiving systemic corticosteroids.

*Group 8*—10 seriously ill patients with non-malignant disease.

These patients were studied in order to assess the effect of debility and advanced metabolic disturbance on tests of monocyte function. Three had terminal pneumonia, 3 severe cardiac failure unresponsive to treatment, 2 advanced cirrhosis of the liver and the remaining 2 cystic fibrosis with marked hypoalbuminaemia, infection and wasting. They were receiving a variety of drugs, but once again patients taking systemic corticosteroids were excluded.

*Group 9*—10 relatives of patients with SCC of the lung.

First-degree relatives, siblings and children of the index patients were studied. They were all healthy at the time of the study and receiving no drug therapy.

*Group 10*—10 patients were re-studied after surgical resection of their primary tumour.

They were investigated no sooner than 6 weeks, and no later than 12 weeks after their operation.

*Groups 11* and *12*—In addition, each patient with cancer was compared with a subject from one of the control groups, matched for sex, age (within the same decade), respiratory function (<50%, 50–75%, >75% of predicted; Cotes, 1975), forced expiratory volume ($FEV_1$) or forced vital capacity (FVC), whichever was least, as assessed by dry spirometry (vitalograph), smoking habit (present smoker, ex-smoker for more than 6 months, non-smoker) and the presence of infection.

Clinical details of all patients and controls are summarized in Table I.

*Monocyte maturation*.—This was based on the assay described by Currie & Hedley (1977). Blood was drawn between 08:30 and 11:00 and the sample divided into 2. The first 30 ml was allowed to clot in sterile plastic universals and the serum separated. Any serum not used the same day was stored at −70°C in 1ml aliquots. The second 30 ml of blood was defibrinated, diluted 1:1 with phosphate-buffered saline (PBS) and layered on a fresh mixture of Ficoll 400 (Pharmacia Fine Chemicals, Uppsala, Sweden) and Triosil 440 (Nyegaard and Company, Oslo, Norway) having a final specific gravity of 1·077. After centrifugation at 500 *g* for 35 min at room temperature, mononuclear cells lying at the interface of serum and Ficoll–Triosil were harvested and washed ×4 in Medium

TABLE I.—*Clinical details of patients with squamous-cell carcinoma of the lung and control groups*

| Group (n) | M:F | Age* | Cigarettes† | $FEV_1$ | FVC | Infected patients‡ |
|---|---|---|---|---|---|---|
| 1. Limited SCC (20) | 18:2 | 63·9 (50–76) | 52·1 (20–100) | 75·1 (25·5–103) | 81·4 (50·4–100) | 4 |
| 2. Extensive SCC (20) | 17:3 | 66·3 (57–81) | 48·2 (6–140) | 65·3 (28–101) | 71·3 (45–100) | 3 |
| 3. Non-smokers (10) | 9:1 | 28·2 (25–35) | — | 98·5 (85–105) | 101·2 (95–104) | — |
| 4. Non-smokers (10) | 8:2 | 63·4 (55–78) | — | 95·7 (82–124) | 98·3 (75–104) | — |
| 5. Smokers (40) | 30:10 | 61·8 (32–75) | 41·7 (15–100) | 97·0 (81–113) | 96·1 (75–110) | — |
| 6. COAD (21) | 17:4 | 66·0 (51–80) | 47·4 (14–100) | 53·4 (21–94) | 66·7 (41–100) | — |
| 7. COAD with infection (12) | 9:3 | 68·0 (49–77) | 44·0 (15–80) | 60·6 (21–101) | 74·6 (25–100) | 12 |
| 8. Terminal NMD (10) | 7:3 | 50·9 (18–78) | 18·2 (0–44) | 53·6 (7·7–98) | 66·2 (26·7–100) | 2 |
| 9. Relatives | 7:3 | 52·6 (32–68) | 15·9 (0–40) | 98·8 (90–110) | 99·6 (83–102) | — |

* Mean years (range).
†Mean pack years (range).
‡ Assessed clinically.
COAD = chronic obstructive airways disease.
NMD = non-malignant disease.
$FEV_1$ = forced expiratory volume in one second (% predicted).
FVC = forced vital capacity (% predicted).

199 (M199, Wellcome, Beckenham). After a final wash in RPMI 1640 (Flow Laboratories, Irvine) supplemented with L-glutamine 2 mM, penicillin 100 u/ml, streptomycin 100 $\mu$g/ml and HEPES buffer (Gibco Europe, Glasgow) to a final concentration of 0·02 M, the cells were counted and adjusted to a final concentration of $4 \times 10^6$/ml in RPMI 1640. Sterile siliconized glassware was used throughout the separation procedure. Only cell preparations with >95% viability by trypan-blue exclusion were used in this study.

Fifty microlitres of cell suspension and 50 $\mu$l of serum were added to the wells of microtitre plates (Nunclon Delta, Denmark) and incubated at 37°C in a humidified atmosphere of 5% $CO_2$ in air. Cytospin preparations (Shandon Southern) were made of the same cell suspensions for histochemical staining. These were fixed with cold buffered formol acetone for 15 sec, air-dried and stained for nonspecific esterase activity (NSE) at pH 6·3, as described by Yam *et al.* (1970). From these preparations, counts of the percentage monocytes were made, a total of 300 cells being counted in each case.

At 7 days each well was washed ×3 with RPMI 1640 to remove non-adherent cells and the nuclei of the remaining adherent cells were released and stained by adding 50 $\mu$l of 0·1M citric acid containing 1:2000 crystal violet (Sanford *et al.*, 1951). After 30 min the solution in each well was mixed vigorously and the number of nuclei counted in a haemacytometer. Monocyte maturation was then expressed as the percentage of monocytes placed in culture at Day 1 which were present as mature adherent macrophages at Day 7. The mean of 5 replicate wells was taken in each case.

Except for relatives and patients with severe non-malignant disease, who were tested once only, all patients and normal controls were tested on two separate occasions within 3 days of each other, and the mean taken.

*Monocyte adherence.*—Two microlitres of mononuclear cells in RPMI 1640 containing 50% autologous serum prepared as above were placed in each of 10 wells of a microtitre plate and incubated for 2 h at 37°C in a humidified atmosphere of 5% $CO_2$ in air. Each well was washed ×3 with RPMI 1640, and the cells adhering to the bottom of each well counted. The mean of 10 wells was taken and the result expressed as a number of cells adherent at 2 h as a percentage of $NSE^+$ cells originally placed in each well.

*Statistical analysis of data.*—The Mann–Whitney U test and the Wilcoxon ranked-pair test were used for comparing differences between groups or changes within groups. The Spearman rank-correlation test was employed for determining the significance of correlations between different measured parameters within a group.

### RESULTS

Preliminary experiments were designed to confirm and extend the findings of Currie and his colleagues (Currie & Hedley, 1977) concerning the characteristics of the maturing cells and the influence of cell numbers, excess concentration and adherence.

The adherent cells present on Day 7 were morphologically identifiable as macrophages by light and electron microscopy. These cells stained avidly for NSE activity, phagocytosed latex particles and demonstrated Fc-receptor activity. Non-adherent cells present at Day 7 were smaller and had the characteristics of lymphocytes. They did not show diffuse NSE staining and were non-phagocytic.

The influence of initial cell concentration on the percentage of cells maturing to macrophages was ascertained by plating serial dilutions of normal mononuclear cells in the wells and determining the numbers maturing by Day 7. Between initial mononuclear cell concentrations of $10^5$/ml and $4 \times 10^6$/ml there was no significant difference in the result obtained for monocyte maturation in a series of 5 normal controls. This suggests that cell density or differences in monocyte numbers within the initial preparations are unlikely to influence maturation results through overcrowding.

Optimum maturation was observed with autologous-serum concentrations between 40 and 65%; we chose to use a concentration of 50% in this study to facilitate practical procedures, and to enable more direct comparison with the results of previous studies.

TABLE II.—In vitro *mononuclear-cell adherence and monocyte maturation*

| Normal controls | Adherence at 2 h (%) | Maturation at 7 days (%) |
|---|---|---|
| 1 | 87·2 | 47·6 |
| 2 | 96·4 | 49·2 |
| 3 | 91·2 | 56·3 |
| 4 | 85·7 | 39·2 |
| Mean | 90·1 | 48·1 |
| SCC patients | | |
| 1 | 76·3 | 7·2 |
| 2 | 72·1 | 23·6 |
| 3 | 78·2 | 6·4 |
| 4 | 91·9 | 24·8 |
| 5 | 87·0 | 36·1 |
| 6 | 82·9 | 15·0 |
| Mean | 81·4 | 17·8 |

Each result is the mean of 5 replicates.

In a series of 6 patients with SCC and 4 normal controls, adherence at 2 h was measured in parallel with a determination of monocyte maturation at Day 7 (Table II). Although cell adherence at 2 h was reduced in some SCC patients, there was no consistent correlation with depressed monocyte maturation at Day 7.

*Variability of results*

The well-to-well variation in results was small, the coefficients of variation of 10 replicate wells being 8·5%, 7·0% and 4·0% in 3 separate sets of cells. The effect of passage of time on results was assessed by measuring the monocyte maturation of one normal control on 20 separate occasions over 3 months. The mean result was 54·8%, with a standard deviation of 6·5% and coefficient of variation 18·0%. Every patient and every control subject in Groups 1–6 was tested on 2 separate occasions within 3 days of each other. The mean difference in percent maturation between the two occasions was 4·4 ± 3·1% for the SCC patients and 5·0 ± 3·8% for the normal controls.

*Monocyte maturation in autologous serum*

A total of 93 controls and 40 SCC patients has been studied. The results of monocyte maturation in Control Groups 3–7 are given in Table III. There was no significant difference that could be attributed to age (Groups 3 & 4), cigarette smoking (Groups 2 & 3) or infection (Groups 6 & 7). There was no significant difference between monocyte maturation in males or females within Groups 3–7, nor any difference between patients suffering from COAD with normal or abnormal respiratory function. For the purposes of further comparison with patients and other control groups the results of all normal controls (Groups 3–5) and all

TABLE III.—In vitro *maturation of monocytes from control groups cultured in autologous serum*

| Group (n) | Monocyte maturation* | *P*† | |
|---|---|---|---|
| Normal controls | | | |
| 3. Non-smokers 25–35 (10) | 47·8 (37·0–62·0) | NS (3 vs 4) | |
| 4. Non-smokers 53–78 (10) | 47·0 (38·2–58·8) | NS (4 vs 5) | |
| 5. Smokers 43–75 (40) | 43·7 (30·4–56·6) | | |
| All male controls (47) | 46·2 (30·5–62·0) | NS (male vs female) | |
| All female controls (13) | 41·5 (30·4–53·0) | | |
| COAD | | | |
| 6. Uninfected (21) | 39·8 (19·7–56·4) | NS (6 vs 7) | |
| 7. Infected (12) | 34·8 (7·6–53·6) | | |
| All COAD: $FEV_1$ or FVC < 50% (14) | 37·1 (7·6–56·4) | NS | NS |
| All COAD: $FEV_1$ or FVC 50–75% (11) | 40·6 (30·4–53·2) | NS | |
| All COAD: $FEV_1$ and FVC > 75% (8) | 35·3 (20·1–56·4) | | |

* Mean % maturation in 50% autologous serum (range).
† Significance of difference between groups assessed by Mann–Whitney U Test.
COAD = chronic obstructive airways disease.
$FEV_1$ = forced expiratory volume in one second.
FVC = forced vital capacity.

FIG. 1.—*In vitro* maturation of monocytes from normal controls, patients with chronic obstructive-airways disease (COAD) and patients with squamous-cell carcinoma (SCC) of the lung. Bars represent means.

FIG. 2.—*In vitro* maturation of monocytes from SCC patients and matched controls (C). Bars represent means.

COAD patients (Groups 6 & 7) have been combined.

The mean monocyte maturation in COAD patients (38·0%) was lower than in normal controls (44·9%) ($P<0{\cdot}05$). Maturation in patients with limited SCC (23·9%) and extensive SCC (14·2%) was lower than in both normal controls ($P<0{\cdot}001$) and COAD patients ($P<0{\cdot}001$) (Fig. 1). As described under Methods, during the study each SCC patient was matched with a relevant subject to control for any possible effect of age, sex, cigarette smoking, infection or respiratory function on monocyte maturation. The results (Fig. 2) show a highly significant difference ($P<0{\cdot}001$) in respect of both limited and extensive disease.

*Relationship between monocyte maturation and clinical state of patients with SCC*

Monocyte maturation in patients with extensive disease was significantly less than in limited disease ($P<0{\cdot}01$). The size of the primary tumour was available in 32/40 SCC patients, either from the chest radiograph or measurement of the surgically removed specimen. An analysis of these patients revealed no correlation between the size of the primary tumour and monocyte maturation.

The effect of debility was assessed in two ways. First, the level of serum albumin in SCC patients was compared with the result of *in vitro* monocyte maturation; no correlation was found. Second, 10 patients with serious terminal non-malignant disease (Group 8) were studied (Table IV). Mean maturation in this group was 19·2%, which was significantly lower than in normal controls ($P<0{\cdot}001$) but very similar to that in the group of patients with limited malignancy. Patients with extensive SCC had lower maturation (14·2%) than those with terminal non-malignant disease (19·2%).

TABLE IV.—In vitro *maturation of monocytes from SCC patients and patients with terminal non-malignant disease (NMD)*

| Group (n) | Serum albumin (g/l) mean ± s.d. | Monocyte maturation* | *P* |
|---|---|---|---|
| Normal controls (60) | 45·1 ± 3·2 | 44·9 (30·4–62·0) | |
| Terminal NMD (10) | 29·3 ± 3·6 | 19·2 (3·6–42·8) | < 0·001† |
| Limited SCC (20) | 39·8 ± 4·0 | 23·9 (3·7–51·6) | { < 0·001†<br>NS‡ |
| Extensive SCC (20) | 34·5 ± 6·5 | 14·2 (2·5–52·9) | { < 0·001†<br>< 0·05‡ |

* Mean % maturation in 50% autologous serum (range).
† Comparison with normal controls.
‡ Comparison with patients with terminal NMD.

TABLE V.—*Surgical resection of primary SCC in* 10 *males: Clinical features, operative findings, clinical course,* in vitro *monocyte maturation*

| Patient | Age (yr) | Size of tumour (cm) | Involvement of hilar nodes | Operation* | Clinical progress† | Monocyte maturation‡ Pre-op. | Post-op. |
|---|---|---|---|---|---|---|---|
| 1 | 60 | 4 × 4 | + | LP | NDD (1 yr) | 18 | 45·3 |
| 2 | 51 | 8 × 8 | − | LUL + BCG | NDD (9 m) | 5 | 31·5 |
| 3 | 64 | 5 × 5 | − | RUL + RML | Met (6 m) | 21·1 | 11·2 |
| 4 | 62 | 2 × 2 | + | RMLL | NDD (6 m) | 51·6 | 48·0 |
| 5 | 64 | 6 × 4 | − | LUL | NDD (7 m) | 31·3 | 22·2 |
| 6 | 67 | 4 × 4 | − | RUL | D (4 m) | 15·3 | 27·4 |
| 7 | 65 | 6 × 6 | + | RUL | NDD (5 m) | 21·7 | 10·0 |
| 8 | 71 | 4 × 4 | + | LP | NDD (1 yr) | 31·3 | 34·8 |
| 9 | 49 | 3 × 5 | − | LUL | NDD (9 m) | 38·5 | 42·0 |
| 10 | 69 | 3 × 5 | − | LUL | NDD (6 m) | 43·0 | 45·8 |

* LP = left pneumonectomy, LUL = left upper lobectomy, RUL = right upper lobectomy, RMLL = right, middle and lower lobectomy; BCG = Tice strain BCG given post-operatively by intrapleural route ($10^7$ viable organisms in 1 ml).

† NDD = no clinically detectable disease; D = died; Met = metastatic liver disease (follow-up period).

‡ Maturation % in 50% autologous serum.

*Genetic influence on monocyte maturation*

To assess the possibility that SCC patients had some genetic predisposition for poor monocyte function, we examined 10 first-degree relatives of SCC patients (Group 9). There was no significant difference between mean maturation in this group, 40·4% (range 34·7–51·7%) and in normal controls, 44·9% (30·4–62·0%).

*Effect of surgical resection on monocyte maturation*

Ten patients were studied longitudinally. Operative findings, clinical course and the comparison of pre- and postoperative monocyte maturation are shown in Table V. The monocyte maturation in normal controls lay within the range 30·4%–62·0%. If values for maturation below 30·4% are considered low, Patients 4, 5, 8, 9 and 10 in Table IV had "normal" preoperative values for monocyte maturation. All 5 patients remained alive without evidence of metastases or locally recurrent disease at least 6 months postoperatively. Postoperatively monocyte maturation was normal in all but Patient 5. The remaining patients had low preoperative maturation and 3 of them also had a low postoperative result. One of these has since died of his disease and another has developed liver metastases. Two patients with low preoperative maturation had normal values after resection (Patients 1 and 2 in Table IV); one of these had received intrapleural BCG immediately after operation as part of a trial of immunotherapy.

### DISCUSSION

The maturation of monocytes into macrophages *in vitro* has been studied for many years (Lewis, 1925) and there is general agreement that *in vivo* maturation of monocytes provides a sizeable proportion of tissue macrophages (Volkman, 1970). Culture of both animal and human monocytes has been used as a source of macrophages suitable for *in vitro* functional studies (Blaese, 1972).

The first indication that maturation of monocytes *in vitro* may be quantitatively altered in patients with abnormalities of the immune system appeared as a comment in a paper by Blaese (1972). He was studying macrophage function using as his source of macrophages monocytes cultured for a period of 2–17 days *in vitro*. He noted that he was unable to study patients with Hodgkin's disease because of persistently very low yields of macrophages. He encountered the same problem with cells from patients with ataxia telangiectasia, in whom multiple defects of the immune system have been reported.

Krikorian (1975) described an assay for macrophage precursors in the blood, and this has been adapted more recently by Currie & Hedley (1977) into a clinically applicable microassay of monocyte maturation. We have been able to confirm some of their findings; namely, higher yields using defibrinated than with heparinized blood, lack of any major variation with differing cell densities over the range used in the assay, and lack of any correlation with adherence of cells at 2 h.

In this study, normal control monocytes matured in a mean percentage of $44{\cdot}9 \pm 6{\cdot}5$, which compares with the normal result ($48{\cdot}3 \pm 19{\cdot}7$) in the paper of Taylor & Currie (1979). The reason for the reduced spread of the results in our study is unclear. Our results also agree with previous work, in finding no effect of age, sex or cigarette smoking on maturation (Currie & Hedley, 1977). We could find no difference between COAD patients with and without clinical infection. This is surprising, because infection is a known stimulus for the outpouring of monocytes and promonocytes from marrow, and the change in number of circulating immature cells might reasonably be expected to alter the number maturing *in vitro*. A possible explanation may be poor identification of patients with infection of sufficient severity. Clinical infection based on history and the presence of purulent sputum is not always indicative of systemic infection, and chronically infected COAD patients do not always have obvious clinical symptoms or signs.

SCC patients are usually smokers and many have coexistent COAD. Because monocyte maturation in COAD patients was significantly less than in normal controls, it was necessary to compare the results in SCC patients with this group or a group matched for the presence of COAD. Both these comparisons were made, and in each case there was significantly depressed maturation in limited and extensive SCC.

Depression of monocyte maturation in 50% autologous serum, found in SCC patients, is similar to that described previously in patients with malignant melanoma (Currie & Hedley, 1977) and with breast carcinoma (Taylor & Currie, 1979). The deficit was more pronounced in those with extensive disease, only 3/20 such patients having values within the range in normal controls, compared with 8/20 with limited disease. This is in keeping with the findings of others (Currie & Hedley, 1977; Taylor & Currie, 1979) that the defect of maturation is quantitatively related to tumour burden. It is too early to comment in any depth on the correlation of monocyte maturation with clinical prognosis, but in respect to the effects of surgery it is perhaps worth noting that of the 2 subjects in whom postoperative maturation was very low, one died after 5 months and the other developed hepatic metastases after 6 months. The other 8 subjects studied longitudinally after surgery remained well at least 6 months later, and this includes 3 patients in whom preoperative monocyte maturation was depressed but returned to normal after resection. One patient who received intrapleural BCG as an adjunct to surgery showed a dramatic increase in maturation from 5% preoperatively to 31·5% 2 months postoperatively. This is of particular interest in view of the dramatic increases in monocyte maturation noted in patients with malignant melanoma after *C. parvum* treatment (Hedley *et al.*, 1979).

The correlation of depression of monocyte maturation with extent of disease raises the question whether increasing metabolic disturbance and general debility underlie the abnormality. Such disturbances may be associated with abnormalities of immune function (Smythe *et al.*, 1971; Law *et al.*, 1973) and macrophage killing of micro-organisms (Olson *et al.*, 1978). However, although this may be partly the cause it is unlikely to be the sole explanation. The group of 10 patients with terminal non-malignant disease was chosen deliberately to represent subjects with severe debility. Their levels of serum albumin were lower than those of the group of patients with extensive malignant disease (Table III), but the latter group showed more depression of monocyte maturation than the former. Within the group of patients with limited and extensive SCC there was no correlation of monocyte maturation with serum albumin level. Furthermore, patients with malignancy were tested at the time of their initial investigation and diagnosis, when they were not severely debilitated.

The possibility that some subjects may be more likely to develop malignancy due to an inherited deficiency of monocyte/macrophage natural surveillance is attractive but we have been unable to show that first-degree relatives of patients with SCC were in any way different from control subjects. Although the number of patients studied was insufficient to exclude genetic influence, the relation of depressed maturation to extent and size of tumour suggests that the defect is secondary to the development of the malignancy.

It is possible that the cause for abnormal monocyte maturation in cancer patients is interference with normal cellular function by soluble factors of host origin, tumour origin, or both. Similar factors have been described previously: for instance, monocyte migration *in vitro* is reduced by serum factors (Maderazo *et al.*, 1978; Kjeldsberg & Pay, 1978), and serum from cancer patients may alter monocyte Fc receptor expression (Rhodes *et al.*, 1979) and inhibit granulocyte-macrophage colony formation (Liu *et al.*, 1979). Our investigation of the possible soluble-factor inhibition of monocyte maturation will be presented in a future communication.

This investigation of monocyte maturation in patients with squamous-cell carcinoma of the lung has confirmed the depression found by other workers in malignant melanoma and breast carcinoma. It extends the spectrum of abnormalities of function found in these cells in the presence of malignancy. This phenomenon may be relevant to the ability of tumour cells to divide and metastasize in a host despite the ability of monocytes and macrophages to kill tumour cells *in vitro*. Unfortunately, this test, like other disturbances of immune function described in the past, does not lend itself to use as a diagnostic aid in early malignancy. In such cases maturation is often normal and when it is abnormal it is likely to be less so than in those with extensive disease. However, it may prove useful in detecting those patients whose disease is judged clinically to be limited, yet who are likely to fare badly. Possibly it is a sensitive marker of tumour load or spread. If so, it may also be a more sensitive monitor of the effect of treatment.

We thank colleague physicians at Brompton Hospital for allowing us to study their patients, particularly Dr Ian Gregg, who found many of the volunteer control subjects.

Dr R. G. Dent was supported by the Brompton Hospital Clinical Research Committee and the work was supported by the Medical Research Council.

The secretarial assistance of Mrs P. Hearn and Miss M. Ash is gratefully acknowledged.

### REFERENCES

BARRETT, O. (1970) Monocytosis in malignant disease. *Ann. Intern. Med.*, **73**, 991.

BLAESE, R. M. (1972) Lymphocyte-mediated interaction in antigen induced lymphocyte transformation in patients with Wiskott-Aldrich disease and other diseases with anergy. *Cell. Immunol.*, **4**, 228.

CHEERS, C. & WALLER, R. (1975) Activated macrophages in congenitally athymic nude mice and thymectomized lethally-irradiated mice. *J. Immunol.*, **115**, 844.

COTES, J. E. (1975) Lung *Function: Assessment and Application in Medicine*. 3rd ed. 1975. Oxford: Blackwell. p. 383.

CURRIE, G. A. & HEDLEY, D. W. (1977) Monocytes and macrophages in malignant melanoma. I. Peripheral blood macrophage precursors. *Br. J. Cancer*, **36**, 1.

DIZON, Q. S. & SOUTHAM, C. M. (1963) Abnormal cellular response to skin abrasion in cancer patients. *Cancer*, **16**, 1288.

EVANS, R. (1972) Macrophages in syngeneic animal tumours. *Transplantation*, **14**, 468.

GORER, P. A. (1961) The antigenic structure of tumours. *Adv. Immunol.*, **1**, 345.

HEDLEY, D. W., NYHOLM, R. E. & CURRIE, G. A. (1979) Monocytes and macrophages in malignant melanoma. IV. Effects of *C. parvum* on monocyte function. *Br. J. Cancer*, **39**, 558.

HIBBS, J. R. (1976) The macrophage as a tumoricidal effector cell: A review of *in vivo* and *in vitro* studies on the mechanism of the activated macrophage nonspecific cytotoxic reaction. In *The Macrophage in Neoplasia*. Ed. Fink. New York: Academic Press. p. 83.

HOLDEN, H. J., HASKILL, J. S., KIRCHNER, H. & HERBERMAN, R. B. (1976) Two functionally distinct anti-tumour effector cells isolated from murine sarcoma virus induced tumours. *J. Immunol.*, **117**, 440.

ISA, A. M. & SANDERS, B. R. (1975) Enhancement of tumour growth in allogeneic mice following impairment of macrophage function. *Transplantation*, **20**, 296.

KING, G. W., LOBUGLIO, A. F. & SAGONE, A. L. (1977) Human monocyte glucose metabolism in lymphoma. *J. Lab. Clin. Med.*, **89**, 316.

KJELDSBERG, C. R. & PAY, G. D. (1978) A qualitative and quantitative study of monocytes in patients with malignant solid tumours. *Cancer*, **41**, 2236.

KREYBERG, L., LIEBOW, A. A. & UEHLINGER, E. A. (1967) *Histological Typing of Lung Tumours*. Geneva: W.H.O. p. 13.

KRIKORIAN, G., MARSHALL, W. H., SIMMONS, S. & STRATTON, F. (1975) Counts and characteristics of macrophage precursors in human peripheral blood. *Cell. Immunol.*, **19**, 22.

LAUDER, I., AHERNE, W., STEWART, J. & SAINSBURY, R. (1977) Macrophage infiltration of breast tumours: A prospective study. *J. Clin. Pathol.*, **30**, 563.

LAW, D. K., DUDRICK, S. D. & ABDOU, N. I. (1973) Immunocompetence of patients with protein-calorie malnutrition. *Ann. Intern. Med.*, **79**, 545.

LEWIS, M. R. (1925) The formation of macrophages, epithelioid cells and giant cells from leucocytes in incubated blood. *Am. J. Pathol.*, **1**, 91.

LIU, Y. K., STALLARD, S., KOO, V. & DANNAHER, C. L. (1979) Serum inhibitor activity of granulocyte-macrophage colony formation in patients with cancer. *Cancer Res.*, **39**, 1640.

MADERAZO, E. G., ANTON, T. F. & WARD, P. A. (1978) Serum-associated inhibition of leukotaxis in humans with cancer. *Clin. Immunol. Immunopathol.*, **9**, 166.

MARGAREY, C. J. & BAUM, M. (1970) Reticulo-endothelial activity in humans with cancer. *Br. J. Surg.*, **57**, 748.

MANTOVANI, A., JERRELS, T. R., DEAN, J. H. & HERBERMAN, R. B. (1979) Cytolytic and cytostatic activity on tumour cells of circulating human monocytes. *Int. J. Cancer*, **23**, 18.

MEDICAL RESEARCH COUNCIL (1965) Definition and classification of chronic bronchitis. *Lancet*, i, 775.

MEURET, G., SCHMITT, E., HAGEDORN, M. & KUHN, A. (1977) Monocyte production in malignant disease. In *The Macrophage and Cancer*. Ed. James *et al.* Edinburgh: p. 438.

OLSON, L. C., SISK, D. R. & IZSAK, E. (1978) Protein-calorie malnutrition impairs the anti-viral function of macrophages. *Proc. Soc. Exp. Biol. Med.*, **159**, 84.

OUTZEN, H. C., CUSTER, R. P., EATON, G. J. & PREHN, R. T. (1975) Spontaneous and induced tumour incidence in germ-free "nude" mice. *J. Reticuloendothel. Soc.*, **17**, 1.

RHODES, J. (1977*a*) Regulatory effects of normal human monocytes and monocytes activated in cancer on normal lymphocyte responses to mitogen. In *The Macrophage and Cancer*. Ed. James *et al.* Edinburgh: p. 390.

RHODES, J. (1977*b*) Altered expression of human monocyte Fc receptors in malignant disease. *Nature*, **265**, 253.

RHODES, J., BISHOP, M. & BENFIELD, J, (1979) Tumour surveillance: How tumours may resist macrophage-mediated host defence. *Science*, **203**, 179.

SANFORD, K. K., EARLE, W. R., EVANS, V. J., WALTZ, H. K. & SHANNON, J. E. (1951) The measurement of proliferation in tissue cultures by enumeration of cell nuclei. *J. Natl Cancer Inst.*, **11**, 773.

SMYTHE, P. M., SCHONLAND, M., BRERETON-STILES, G. G. & 6 others (1971) Thymolymphatic deficiency and depression of cell-mediated immunity in protein-caloric malnutrition. *Lancet*, ii, 939.

SNYDERMAN, R., SEIGLER, H. F. & MEADOWS, L. (1977) Abnormalities of monocyte chemotaxis in patients with melanoma: Effects of immunotherapy and tumour removal. *J. Natl Cancer Inst.*, **58**, 37.

TAYLOR, S. A. & CURRIE, G. A. (1979) Monocyte maturation and prognosis in primary breast cancer. *Br. Med. J.*, i, 1050.

TEVETHIA, S. S., ZARLING, J. M. & FLAX, M. H. (1976) Macrophages and the destruction of syngeneic virus-induced tumours. In *Immunobiology of the Macrophage*. Ed. Nelson. New York: Academic Press. p. 509.

VOLKMAN, A. (1970) The origin and fate of the monocyte. *Sem. Haematol.*, **3**, 62.

VOSE, B. M. (1978) Cytotoxicity of adherent cells associated with some human tumours and lung tissues. *Cancer Immunol. Immunother.*, **5**, 173.

YAM, L. T., LI, C. Y. & CROSBY, W. H. (1970) Cytochemical identification of monocytes and granulocytes. *Am. J. Clin. Pathol.*, **55**, 283.